# A clinicopathological study of selected cognitive impairment cases in Lothian, Scotland: enhanced CJD surveillance in the 65 + population group

**DOI:** 10.1186/s12877-022-03280-4

**Published:** 2022-07-20

**Authors:** Lovney Kanguru, Gemma Logan, Briony Waddel, Colin Smith, Anna Molesworth, Richard Knight

**Affiliations:** 1 National CJD Research & Surveillance Unit (NCJDRSU), University of Edinburgh, Western General Hospital, Edinburgh, Scotland; 2 NHS Lothian and Queen Margaret University, Edinburgh, Scotland; 3 Department of Neurology, Ninewells Hospital, Dundee, Scotland; 4 Edinburgh Brain Bank (EBB), Centre for Clinical Brain Sciences, University of Edinburgh, Chancellor’s Building, 49 Little France Crescent, Edinburgh, Scotland; 5 Public Health Scotland (PHS), Glasgow, Scotland

**Keywords:** Creutzfeldt-Jakob Disease, vCJD, sCJD, Prion disease, Surveillance, Public health, Health protection, Scotland, Geriatric, Neurology

## Abstract

**Background:**

Variant Creutzfeldt-Jakob Disease (vCJD) is primarily associated with dietary exposure to bovine-spongiform-encephalopathy. Cases may be missed in the elderly population where dementia is common with less frequent referral to specialist neurological services. This study’s twin aims were to determine the feasibility of a method to detect possible missed cases in the elderly population and to identify any such cases.

**Methods:**

A multi-site study was set-up in Lothian in 2016, to determine the feasibility of enhanced CJD-surveillance in the 65 + population-group, and undertake a clinicopathological investigation of patients with features of ‘atypical’ dementia.

**Results:**

Thirty patients are included; 63% male, 37% female. They were referred because of at least one neurological feature regarded as ‘atypical’ (for the common dementing illnesses): cerebellar ataxia, rapid progression, or somato-sensory features. Mean-age at symptom-onset (66 years, range 53–82 years), the time between onset-of-symptoms and referral to the study (7 years, range 1–13 years), and duration-of-illness from onset-of-symptoms until death or the censor-date (9.5 years, range 1.1–17.4 years) were determined. By the censor-date, 9 cases were alive and 21 had died. Neuropathological investigations were performed on 10 cases, confirming: Alzheimer’s disease only (2 cases), mixed Alzheimer’s disease with Lewy bodies (2 cases), mixed Alzheimer’s disease with amyloid angiopathy (1 case), moderate non-amyloid small vessel angiopathy (1 case), a non-specific neurodegenerative disorder (1 case), Parkinson's disease with Lewy body dementia (1 case), and Lewy body dementia (2 cases). No prion disease cases of any type were detected.

**Conclusion:**

The surveillance approach used was well received by the local clinicians and patients, though there were challenges in recruiting sufficient cases; far fewer than expected were identified, referred, and recruited. Further research is required to determine how such difficulties might be overcome. No missed cases of vCJD were found. However, there remains uncertainty whether this is because missed cases are very uncommon or because the study had insufficient power to detect them.

## Introduction

Prion diseases affect animals and humans and, in humans, exist in idiopathic (sporadic), genetic, and acquired forms. Variant Creutzfeldt-Jakob Disease (vCJD) is a very rare, acquired form of human prion disease, which arose as a zoonosis associated with dietary exposure to bovine spongiform encephalopathy (BSE; a prion disease of cattle). Secondary human to human transmission has occurred via blood (red cell transfusion and factor VIII treatment) [1, 2], but no other transmission routes, such as surgery and dentistry, have been identified [3, 4]. Despite an estimated significant dietary exposure before UK control measures were instituted between 1988 and 1996 [5, 6], only 178 cases of definite or probable vCJD have occurred in the UK with no vCJD deaths reported since 2016 [7]. The fact that relatively few clinical cases have resulted from a potentially large exposure has several possible explanations. Firstly, it is likely that there is a significant species transmission barrier between bovine and man. Secondly, when prion diseases are acquired, there is often a long incubation period (up to 40 years) [8]; in the context of BSE and vCJD, the mean is estimated to be up to 10 years [9, 10]. Susceptibility to disease and incubation period length are related, at least in part, to a polymorphism in the human prion protein gene (*PRNP*). At *PRNP-*codon 129, there are 3 common variations resulting in MM, MV, or VV corresponding protein types; about 37% of the UK population is MM, 51% MV and 12% VV [11]. Studies of sporadic (sCJD), kuru and iatrogenic CJD indicate that MM individuals are more susceptible to disease [5, 12–14] and, in acquired cases, with a shorter incubation period [15]. This suggests that MM individuals would be expected to be more susceptible to BSE infection and to have the shortest incubation period. All but one of the UK vCJD cases with genetic testing have been of the MM genotype [16]. Overall, further vCJD clinical cases are expected but in uncertain numbers and timing [9]. There is experimental transmision evidence to support the view that MV and VV individuals are less susceptible to vCJD and have longer incubation periods if they develop disease [15]. The same study suggests that some BSE infection might be truly subclinical i.e. infection never resulting in clinical disease. Therefore, there are good grounds for thinking that currently asymptomatic infected individuals exist in the UK population who may be capable of infecting others; a vital public health consideration. Since abnormal prion protein is found in lymphoreticular tissue in vCJD and is present in the preclinical phase, studies have been performed on routine surgical tonsil and appendix samples [17–19]. On the basis of these studies, and the assumption that any detected abnormal prion protein reflects BSE infection, the UK population prevalence of asymptomatic vCJD is estimated to be 1 in 2000, although one recent study has suggested that this assumption might be problematic [20]. The estimated prevalence of asymptomatic infection contrasts with the small number of both clinical cases and secondary transmission events. One potential explanation is case under-ascertainment. Arguably under-ascertainment might occur particularly in the elderly, where other commoner dementing brain diseases are common, sCJD (an important differential diagnosis of vCJD) is mainly found, with some evidence that the vCJD clinical phenotype might be different in older patients than in younger ones, autopsy rates are low and specialist neurological referral less frequent [21, 22]. With the overlap in clinical phenotypes of prion diseases, the best ascertainment of vCJD requires ascertainment of all forms of human prion disease, so this study aimed to identify cases of all CJD types. Although the primary study aim was to detect unsuspected cases of vCJD, there was a possible additional outcome of interest. The age-specific mortality rate for sCJD falls off after the age of 80 years [16] whereas current theories of aetiology could argue for a continually increasing incidence with age. Therefore, there is the possibility that there might be underascertainment of elderly sCJD cases especially given generally low autopsy rates [22].

In 2016, a multi-site study was set up in Lothian to determine the feasibility of enhanced CJD surveillance in the 65 + population and, undertake a clinicopathological investigation of patients with atypical features of dementia accessing psycho-geriatric services, thereby, detect otherwise unrecognised prion disease.

A study to ascertain possible under-ascertainment of vCJD cases in the young was based on reporting and reviewing all cases of such deterioration in UK children, however this was possible because the relevant clinical presentation was relatively uncommon [23–25]. A similar process would be numerically impossible in the elderly and so some selective criteria (clinical and geographical) were defined, as detailed in the methods section below.

## Methods

### Base population and sample size

In general, the incidence of neurodegenerative diseases causing dementia increases with age, particularly after the age of 65. Latest UK estimates show around 850,000 people suffer from dementia [26]; the most common diagnoses being Alzheimer’s Disease (AD) and/or vascular dementia (VD), and less frequently, dementia with Lewy bodies (DLB), frontotemporal dementia (FTD), Parkinson’s disease (PD) and other neurodegenerative diseases. The majority of these conditions can be diagnosed with reasonable confidence due to their characteristic presentations and some investigation results, however, around 10% of patients [27] may present atypically with unusual features or lacking characteristic ones, with consequent diagnostic uncertainty; missed cases of prion disease might be found particularly in this group.

In Lothian, 125,000 adults are over 65 years [28]. Of these, around 1% are expected to develop dementia annually [26], of which, around 10% might be considered clinically ‘atypical’. Considering an 80% power for detection of cases with 95% confidence intervals within a 5% margin of error, a sample size of 100 cases/year was deemed appropriate. However, it is difficult to estimate cases that would become known to dementia services out-with primary care and, therefore potentially identified as eligible to join the study.

### Eligibility (Table 1)

**Table 1:** Eligibility criteria

	**Inclusion Criteria**	**Exclusion Criteria**
Cases	• Patients aged 65 years or above • Patients who have features considered clinically ‘atypical’ or unusual for the recognised forms dementia e.g. cerebellar ataxia, rapid progression, and sensory features	• Patients below 65 years at the time of referral • Patients diagnosed with a clear alternative pathology e.g., positive diagnostic genetic tests for a known inherited dementia (excluding prion disease); a clear psychiatric diagnosis; space-occupying lesions; neuroinflammatory or neuroinfectious conditions; a history and documented radiological evidence of a cerebral insult temporally related to the onset of symptoms
Site/Specialty clinics in NHS Lothian	• Ann Rowling Clinic (ARC) • Neurology • Psychiatry of old age, • Medicine of the elderly	• Specialised dementia clinics out-with the NHS Lothian Health Board • All other specialty clinics except those in the inclusion criteria

#### Cases

Patients aged 65 + with ‘atypical’ features defined as presentations thought, by the referring clinicians, to be different from what they regarded as characteristic for AD, VD, LBD, FTD, and PD, were eligible. New and existing patients with ‘atypical’ features e.g. rapid disease progression, cerebellar ataxia, and somatosensory features, were eligible.

Exclusion: non-prion pathological diagnosis; known inherited non-prion dementia; clear psychiatric diagnosis; space-occupying brain lesion and neuroinflammatory conditions; radiological evidence of a cerebral insult temporally related to symptoms.

#### Sites

Eligible sites included psychiatry of old age, medicine of the elderly, and neurology (including Ann Rowling Clinic (ARC) specialties, all of which provide dementia services. Sites providing dementia services outside NHS Lothian were excluded.

### Recruitment and follow-up

**Fig. 1:**
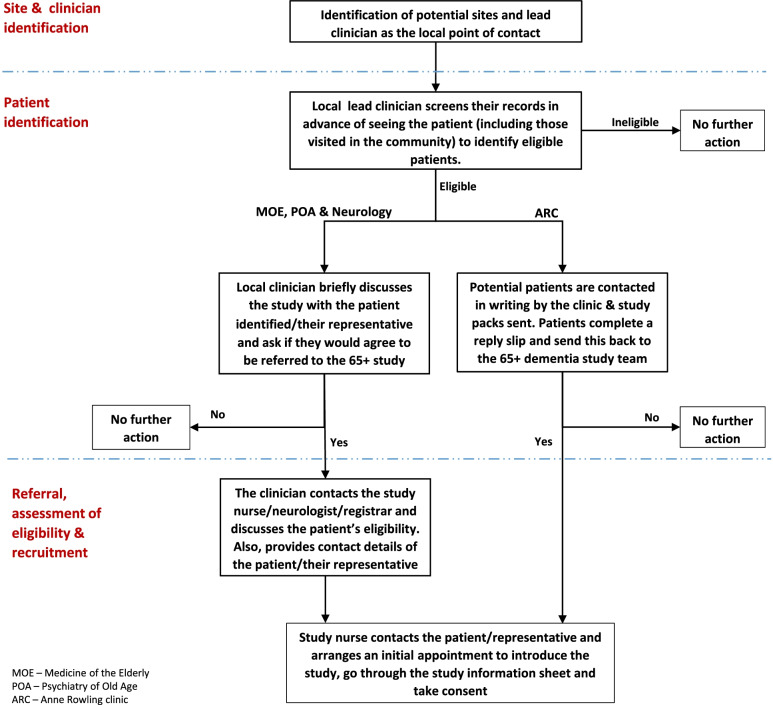
Recruitment process

Figure 1 shows the recruitment process. First, an eligible site was identified. Second, a lead clinician was identified as the point of contact who ascertained eligible patients. To protect patient confidentiality, the lead clinician ensured suitability of the patient by checking the patient's records against the study’s eligibility criteria. For those deemed eligible, the clinician briefly discussed the study with the patient if they had capacity, and/or with their representative, and gave them the 65 + study pack: patient study information sheet, consent form and reply slip. For consented patients, the local clinician contacted the study team to countercheck eligibility and referral was made. At the ARC, the lead clinician screened their database to identify eligible patients. Those identified were contacted in writing and the study pack provided. The study nurse arranged a meeting with those who responded.

All the included cases were followed up initially by telephone within 1 month of recruitment, subsequently, face-to-face every 3 months, and monthly via TraKcare: a comprehensive hospital care management system that records admissions, discharges, and the care provided. From time to time, the clinical research registrar reviewed the cases for evidence of prion disease. Follow-up was until resolution of symptoms or death, but discontinued if a patient/representative withdrew. An email update was sent to the Lothian clinician network forum monthly.

### Data collection and investigations

Demographic and referral information was collected. The onset of symptoms was determined as the year of first self-reported clinical symptoms; verified (as much as possible) with the general practitioner’s notes. Referral was noted as the date the case was referred.

Age at onset of symptoms, suspected diagnoses, duration of illness (time between age at onset of symptoms and death or censor date), and past medical, surgical, blood transfusion, family history of CJD and dementia, occupation, and residential histories, were collected.

A combination of specific neurological assessments were done: the Addenbrooke’s Cognitive Examination (ACE-III) [29], the Severe Impairment Battery—Short Form (SIB-S) [30], the MRC Scale [31], a Frontal Assessment Battery (FAB) [32], Hospital Anxiety and Depression Scale (HADS) [33], and the Edinburgh Motor Assessment (EMAS) Scale [34]. Brain Magnetic Resonance Imaging (MRI) scan was offered to all cases as part of their diagnostic work-up, if this had not already been undertaken. For those who had not had recent MRI scans, arrangements were made for a research MRI brain scan at the University of Edinburgh Brain Research Imaging Centre (BRIC), which involved a Diffusion Weighted Imaging (DWI) and fluid-attenuated inversion recovery (FLAIR) (2D or 3D) as part of a standard brain protocol.

If the results evidenced prion disease, the referring clinician was alerted, who then referred the case to the National CJD Research & Surveillance Unit (NCJDRSU). Blood (2 ml) or buccal samples to determine *PRNP*-129 polymorphism, were collected. None of the cases had a lumbar puncture for Cerebral Spinal Fluid (CSF) tests. These would have been undertaken if indicated by usual clinical criteria/practice, including a strong clinical suspicion of CJD, but they were not part of the research-led investigations.

The possibility of brain tissue donation was raised in life with the patient/representative. In the event of death, where consent was given, a limited, head-only autopsy was arranged. Standard neurological disease histopathology was conducted: sections of tissue from each of the four cortical regions (frontal, temporal, occipital, parietal), the thalamus and the cerebellum were retained,fixed in formalin and processed to paraffin blocks. Each of the six samples was subjected to a standard suite of investigations for neurodegenerative disease using a panel of markers for neurodegenerative proteins (tau, β-amyloid, α-synuclein, p-TDP-43) and screening for spongiform change. Abnormal prion protein was assessed by immunohistochemistry (12F10 and KG9 antibodies) and by biochemistry. Biochemical analysis required the use of approximately 2-3 g of frozen tissue from each of the frontal, temporal, occipital and parietal regions, the thalamus and the cerebellum. Investigations included the most sensitive abnormal prion protein detection methods currently available, which might offer advantages over routine diagnostic techniques in a presumed negative population. These were conducted in series on all samples as follows: standard diagnostic Western blot (WB) for protease-resistant prion protein (PrP^res^) [35], High sensitivity sodium phosphotungstic acid precipitation(NaPTA) WB for PrP^res^ [36], Conformation dependent immunoassay (CDI) analysis for PrP^Sc^ [37], Single round protein misfolding cyclic amplification (PMCA) for ultra-sensitive vCJD PrP^Sc^ detection [38] and real-time quaking induced conversion (RT-QuIC) for ultra-sensitive sCJD PrP^Sc^ detection [39]. The details of these tests are not included in this paper (but there were no findings suggestive of CJD, including vCJD).

### Outcome measures

First, to establish whether the approach used for enhanced CJD surveillance in the 65 + population was feasible, i.e. whether one could recruit suitable patients in sufficient numbers and enhance their assessment, investigation, and diagnosis. Using Bowen’s framework, acceptability, demand, practicality, integration, and efficacy were assessed [40]. Effort expended (staff involved versus numbers recruited), cases expected versus identified, recruitment rate, and proportions consented to clinical-investigations and autopsy were also examined.

Second, to describe the clinicopathological characteristics of the cases to identify whether there were any previously unsuspected prion diseases, and, if so, to determine why they might not be identified in usual clinical practice.

## Results

Recruitment occurred between 1^st^ April 2016 and 30^th^ June 2019, with ongoing follow-up to date. Table 2 summarises the characteristics of cases.

**Table 2:** Cases characteristics

**Demographic characteristics **	**N**	**%**
**Total cases**	**30**	**(100%)**
**Sex**		
Male	19	(63%)
Female	11	(37%)
**Age at study referral**		
65 – 69	9	(30%)
70 – 74	8	(27%)
75 – 79	8	(27%)
80 +	5	(16%)
**Status**		
Alive	9	(30%)
Dead	21	(70%)
**Referral characteristics**	N	%
**Referral specialty**		
Ann Rowling Clinic	15	(50%)
Neurology	4	(13%)
Psychiatry of Old Age	8	(27%)
Medicine of the Elderly	3	(10%)
**Interval between onset of symptoms and referral to the study**	Mean = 7 years (1 – 13 years, std dev 4.1)	
*By age group*		
65 – 69	Mean = 8 years (2 – 13 years, std dev 4.5)	
70 – 74	Mean = 6 years (3 – 11 years, std dev 3.4)	
75 – 79	Mean = 8 years (1 – 13 years, std dev 4.5)	
80 +	Mean = 5 years (2 – 10 years, std dev 3.3)	
**Clinical characteristics**	N	%
**Age at onset of symptoms**	Mean = 66 years (53 – 82 years, std dev. 7.8)	
**Suspected diagnosis**		
Atypical Alzheimer's Disease	11	(37%)
Frontotemporal dementia	4	(13%)
Mixed ^a^	6	(20%)
Unclear/unknown	9	(30%)
**CJD-consistent features **^**b**^		
Cerebellar ataxia	14	(47%)
Involuntary movements	8	(27%)
Rapid progression	7	(23%)
Sensory features	2	(7%)
Visual disturbance	1	(3%)
**Duration of illness (from the onset of symptoms to censor date/death)**	Mean = 9.5 years (1.1 – 17.4 years, std dev 4.7)	
**Symptoms**		
a) Forgetfulness/ memory impairment	28	(93%)
b) Visuospatial difficulties (in recognising objects, familiar people, reading difficulties)	22	(73%)
c) Executive dysfunction (impaired problem solving/judgement)	29	(97%)
d) Language disturbance		
Receptive	18	(60%)
Expressive	26	(87%)
e) Psychiatric symptoms		
Depression	24	(80%)
Anxiety	19	(63%)
Delusions	6	(20%)
Behavioural disturbance	20	(67%)
Impulsive/socially inappropriate	14	(47%)
Change in food preferences	12	(40%)
Aggressive	10	(33%)
Lacking in empathy	14	(47%)
Repetitive movements/behaviours	17	(57%)
Apathy/ withdrawal	21	(70%)
f) Hallucinations		
Visual	8	(27%)
Audio	3	(10%)
g) No insight	16	(53%)
h) Disturbance of gait	21	(70%)
i) Bedbound	3	(10%)
j) Dysarthria	10	(33%)
k) Visual Impairment	3	(10%)
l) Fluctuations of symptoms	3	(10%)
m) Weakness of limbs	6	(20%)
n) Clumsiness of limbs	9	(30%)
o) Slowness of movement	18	(60%)
p) Apraxia	23	(77%)
q) Involuntary movements		
Tremor	18	(60%)
Jerking	12	(40%)
Dystonia	0	0
Alien limb	1	(3%)
r) Seizures	4	(13%)
Akinetic mutism	0	0
t) Sensory symptoms		
Numbness/tingly/paraesthesia	5	(17%)
Pain/burning/discomfort)	3	(10%)
**Other relevant histories**	N	%
**Surgical history (including transplant)**		
No surgery	2	7%
1 – 2	10	33%
3 – 4	9	30%
5 +	9	30%
**Blood transfusion history **^**c**^		
No	29	97%
Yes	1	3%
**Previous injections **^**d**^		
No	24	80%
Yes	6	20%
**Family history of dementia**		
No	14	47%
Yes	13	43%
Unsure/unclear/not known	3	10%
**Occupation history**		
High-risk occupations ^e^	7	23%
Other	23	77%

^a^ mixed: early-onset Alzheimer’s Disease / vascular dementia (3 cases); Corticobasal syndrome/ Progressive supranuclear palsy (1); Frontotemporal dementia /vascular (1)

^b^ some cases presented with more than one feature

^c^ including blood components or plasma products e.g., immunoglobulins or albumins

^d^ as part of treatment e.g., human growth hormone, human gonadotrophin, insulin, fertility treatment

^e^ included: healthcare professions in medical, dentistry, nursing, and paramedical. Laboratory-based professions that dealt with animals, pharmaceutical, or other related research. Professions in veterinary medicine, animal farming, and the meat industry

*Note*: Std dev is standard deviation

### Demographics and referral

Thirty-one cases were recruited, 1 withdrew, thereby, only 30 cases are considered. The majority were male (63%), and most were below 80 years (84%). Fifty-percent of the referrals came from the ARC. On average, the interval between onset of symptoms and referral was 7 (range 1 – 13) years; with the oldest age group (80 + years) having a relatively short interval on average, of 5 years (range 2 – 10 years). By the censor date (28.02.2021), 70% of the cases had died.

### Clinical characteristics

The mean age at onset of symptoms was 66 (range 53 – 82) years. Thirty-seven percent were referred with a suspicion of atypical AD, 30% had unclear/unknown dementia, 20% had mixed dementia, and 13% had FTD. All the cases exhibited at least one ‘atypical’ neurological feature. The mean duration of illness was 9.5 (range 1.1 – 17.4) years.

A range of other clinical symptoms were also noted. All except one case had executive dysfunction (97%). Other common symptoms included: forgetfulness/ memory impairment (93%), language disturbance (expressive (87%) and receptive (60%)), visuospatial difficulties (73%), disturbance in gait (70%), apraxia (77%), slowness of movement (60%) and lack of insight (53%). Psychiatric symptoms were also noted, of which, 80% presented with depression, 70% apathy/ withdrawal, 67% had behavioural disturbances, 63% had anxiety, and 57% had repetitive movements/behaviours. Involuntary movement particurlarly tremor was seen in 60% of the cases. Only 8 cases had sensory symptoms, of which, 5 had numbness/ tingly/ paraesthesia and the remaining 3 had pain/ burning/ discomfort. No case presented with dystonia or akinetic mutism.

### Other relevant histories

The majority of cases had at least 1 previous surgical intervention (93%) before referral. Examining the 3-5 year pre-and-post operative period showed no link with cognitive decline. Similarly, no evidence was found between blood products or previous injections (treatments) and cognitive decline. About two-fifths had a family history of dementia, and none, of prion disease. A small proportion worked in professions considered at risk for prion disease, but no evidence of a link was found.

### Brain MRI Investigation

Twenty-one cases (70%) consented to new or review of previous brain MRI (Table 3). A wide variation between when the brain MRI was performed and the date of onset of first symptoms was observed (19 days—3,626 days (around 10 months)). All the brain MRIs reviewed were found to have some abnormalities. The most common abnormality identified was atrophy: 6 cases had global/generalised cerebral atrophy (1 case was exaggerated in occipital lobes, 2 cases had moderate atrophy (advanced for age), 1 case also had supatentonial small vessel disease change though static since 2013, and 2 cases were generally noted to have cerebral atrophy advanced for age); 8 cases had focal atrophy/ biparietal volume loss (affecting the temporal, parietal, or occipital lobes) and 1 case was described to have generalised atrophy (unspecified). Microvascular ischaemia/ chronic small vessel disease changes were also noted in 3 cases: 2 cases had moderate microvascular ischaemia, and 1 case, advanced chronic small vessel disease with extensive atrophy in the medial temporal lobes. Two cases had diffuse prominence of the ventriculosulcal system which was advanced for age: 1 case had moderate diffusion and focal atrophy in the left temporal lobe and peri-insular cortex, and the second case had marked diffusion more prominent in temporal and parietal lobes bilaterally. None of the abnormalities identified was suggestive of prion disease, therefore, further clinical investigations were not undertaken.

**Table 3:** Brain MRI Investigation Results

Case	Date of onset^a^	MRI date	Period ^~^ (days)	Summary of abnormality
ID 1	01/12/2012	07/08/2013	249	Generalised cerebral atrophy, exaggerated in occipital lobes
ID 2	15/07/2013	15/09/2015	792	Moderate global cerebral atrophy, accelerated for age. Minor small vessel change with no focus on ischaemic change
ID 3	15/07/2009	31/10/2013	1569	Moderate diffuse prominence of the ventriculosulcal system, slightly more prominent in the frontal and parietal lobes bilaterally and slightly advanced for age. Patchy hyper intense FLAIR signal present throughout the cerebral deep white matter. Global cerebral atrophy. More focal atrophy left temporal lobe and peri-insular cortex
ID 4	15/07/2011	28/04/2014	1018	Marked diffuse prominence of the ventriculosulcal system, advanced for stated age. More prominent in temporal and parietal lobes bilaterally
ID 5	15/07/2004	19/06/2014	3626	Generalised cerebral atrophy, probably slightly advanced for age
ID 6	15/07/2011	21/09/2016	1895	Mild bilateral temporal lobe atrophy, worse on the left. No features of prion disease
ID 7	20/10/2015	12/08/2016	297	Moderate microvascular ischaemia probably more recent punctate cortical infarction in the high right parietal lobe
ID 8	15/07/2010	06/10/2016	2275	Subtle biparietal volume loss in keeping with Alzheimer's type dementia. No features of prion disease
ID 9	15/10/2013	13/03/2014	149	Cerebral atrophy, advanced for age
ID 10	15/07/2003	06/04/2009	2092	Mild degree of generalised atrophy for age but otherwise examination unremarkable
ID 11	01/12/2015	17/06/2017	564	No imaging features of prion disease. Global atrophy, with more focal atrophy in perisylvian cortex bilaterally and left medial temporal lobe structures. Enlarged lateral and third ventricles are most likely secondary to atrophy
ID 12	15/07/2014	16/12/2016	885	Marked medial temporal lobe atrophy supporting diagnosis of AD. Non-specific white matter changes most likely secondary to small vessel disease
ID 13	15/07/2013	10/05/2017	1395	No MR features of CJD. Advanced global atrophy with more marked medial temporal atrophy
ID 14	17/08/2011	11/11/2014	1182	Generalised cerebral atrophy advanced for age. Supatentonial small vessel change static since 2013. No specific imaging features to indicate PSP
ID 15	15/07/2012	07/06/2015	1057	Moderate global cerebral atrophy for age. No other specific causes for cognitive impairment identified
ID 16	06/09/2012	25/09/2012	19	Generalised involutional change slightly excessive for age but no focal atrophy. No features of CJD. Posterior/medial temporal pattern of atrophy in keeping with a posterior dementia such as Alzheimer’s
ID 17	15/07/2015	14/06/2017	700	Advanced chronic small vessel vascular changes. Extensive atrophy, most evident in the medial temporal lobes
ID 18	30/01/2017	26/04/2017	86	Moderate volume loss and microvascular ischaemia
ID 19	15/07/2014	12/10/2015	454	Cerebral white matter and basal ganglia small vessel ischaemic changes, slightly prominent even allowing for age. Moderate generalised cerebral involution, slight more prominent in temporal lobes. Mixed vascular and neurodegenerative aetiology likely
ID 20	28/06/2012	16/10/2013	475	Asymmetrical atrophy, slight progression since 2009
ID 21	21/03/2017	20/04/2017	30	Bilateral parietal volume loss

^a^ The date of first self-reported clinical symptom(s); verified (as much as possible) with the general practitioner’s notes. ^~ ^Time period between date of onset and MRI date

### Genotyping

Eighty-three percent of the cases consented to *PRNP-*129 genotyping (Table 4): 32% were MM, 52% MV and 16% VV. Carriers of the MM genotype had a shorter duration of illness (8.3 years) than MV (10.4 years) or VV (11.3 years). On average, age at onset of symptoms had minimal variation across the 3 genotypes (MM 64 years, MV 65 years and VV 63 years).

**Table 4:** Genotyping

	**N**	**%**
**Genotyping (*PRNP*-Codon 129)**		
Study consented	25	83%
*Of whom tested*	25	100%
MM	8	32%
MV	13	52%
VV	4	16%
*Duration of illness* (from the onset of symptoms to censor date/death)		
MM	Mean = 8.3 years (1.1 – 16.6 years, std dev 5.5)	
MV	Mean = 10.4 years (4.1 – 17.4 years, std dev 4.6)	
VV	Mean = 11.3 years (6.6 – 16.6 years, std dev 4.7)	
*Age at onset of symptoms*		
MM	Mean = 64 years (56 – 79 years, std dev 7.4)	
MV	Mean = 65 years (53 – 78 years, std dev 7.8)	
VV	Mean = 63 years (56 – 69 years, std dev 5.4)	
*Gender*		
MM	Male = 5 (63%), Female = 3 (37%)	
MV	Male = 8 (62%), Female = 5 (38%)	
VV	Male = 2 (50%), Female = 2 (50%)	
Not known	Male = 4 (80%), Female = 1 (20%)	

*Note*: Std dev is standard deviation

### Autopsy neuropathology

Sixty-four percent consented to autopsy neuropathology examination (Table 5). Unfortunately, delays in notification where embalming had taken place and COVID-19 restrictions meant autopsy could not be performed for some cases and was only available for 10 cases. All cases were assessed using validated grading-and-staging systems, and where a neuropathological diagnosis of AD-neuropathological change (AD-NC) was made, a final clinicopathological diagnosis was made based on the current National Institute on Aging-Alzheimer’s Association (NIA-AA) criteria (PMID: 22,265,587). The final pathological diagnosis in the cases examined was: Alzheimer’s disease only (2 cases); mixed Alzheimer’s disease with Lewy body dementia (2 cases); Alzheimer’s disease with severe cerebral amyloid angiopathy (1 case); vascular dementia with non-amyloid small vessel angiopathy (1 case); Lewy body dementia (3 case). No prion disease cases were detected. One case could not be characterised either clinically or pathologically; a rapidly progressive neurodegenerative disorder clinically thought to be frontotemporal dementia although with late onset cerebellar ataxia. No significant pathology was seen with immunohistochemical analysis of tau, β-amyloid, α-synuclein, FUS, p62, polyQ [trinucleotide repeat diosrders], and both 12F10 and KG9 [prion disorders].

**Table 5:** Autopsy Neuropathology

				**N**		**%**
Study consented				22		(73%)
* of whom deceased*				14		(64%)
* of which performed*				10		(71%)
**Summary of cases (10)**						
	**Codon 129**	**Suspected clinical diagnosis**	**Atypical features noted**		**Autopsy neuropathology findings**	
**ID 1**	MM	Senile dementia, ? Alzheimer’s disease	Fluent dysphagia (very early on), extreme behavioural disturbance		Alzheimer’s Disease- Neuropathological changes (AD-NC), neocortical Lewy body, severe cerebrovascular disease	
**ID 2**	MV	Early-onset Alzheimer’s disease, mixed vascular	Seizures		AD-NC	
**ID 3**	MM	Early-onset Alzheimer’s disease	Rapid deterioration, balance impaired		AD-NC, Lewy body dementia	
**ID 4**	MV	Early-onset Alzheimer’s disease	Frontal features, slow progression		AD-NC	
**ID 5**	MM	Early-onset Alzheimer’s or vascular dementia	Unclear		AD-NC, severe cerebral amyloid angiopathy, moderate non-amyloid small vessel disease	
**ID 6**	MM	Early-onset frontal–temporal dementia	Unusual rate of progression		Non-specific neurodegenerative disorder^a^	
**ID 7**	MM	? vascular dementia with behavioural and psychological symptoms of dementia (BPSD)	Considered previously to have a mild cognitive impairment, but evolved quickly to dementia with BPSD		Vascular dementia, microinfarcts, lacunar infarcts	
**ID 8**	Not determined	Complex syndrome, parkinsonian features	? Parkinsonism in dementia, hallucinations. Did not meet the criteria for Lewy body dementia, frontal–temporal dementia, vascular dementia with parkinsonism		Parkinson’s disease, neocortical Lewy body dementia	
**ID 9**	MV	Progressive supranuclear palsy	Possible progressive supranuclear palsy, but insufficient evidence to fulfil diagnostic criteria		Neocortical Lewy body disease limbic predominant age-related TDP43 encephalopathy (stage 3)	
**ID 10**	Not determined	Alzheimer’s disease, mixed vascular dementia	Mixed Alzheimer's or vascular dementia, rapid decline, parkinsonian features		Lewy body dementia	
Age at onset of symptoms			Mean = 65.4 years (56 – 79 years, std dev 7.1)			
Interval between onset of symptoms and referral to the study			Mean = 8 years (2 – 13 years, std dev 3.8)			
Duration of illness (from the onset of symptoms to death)			Mean = 9.9 years (3 – 16 years, std dev 4.4)			

^a ^Could not be characterised either clinically or pathologically; a rapidly progressive neurodegenerative disorder clinically thought to be frontotemporal dementia although with late onset cerebellar ataxia

### Feasibility

The study was well-received, accepted, regarded as appropriate, and perceived to have a positive effect by both patients/representatives and clinicians. The study was seen to provide further clarity on difficult diagnoses. A number of clinicians expressed their intention to refer cases that fitted the study’s criteria. The study was perceived to fit well (to some extent) within the existing dementia services. Despite significant efforts involving several staff (research nurse, clinical research registrar, study neurologist, neuroradiologist, neuropathologist, two post-doctoral laboratory researchers, and an epidemiologist), recruitment fell well below the predicted eligible numbers. It was anticipated that 300 cases might be recruited with only 30 cases recruited (28% of 109 who met the eligibility criteria from 128 potential cases identified and referred). Retention was, however, good with only 1 withdrawal. All the recruited cases underwent clinical examination but only 70% consented to brain MRI investigation or a review of their previous brain MRI investigation report. Only 10 out of the 21 cases that died, underwent autopsy neuropathology examination.

### Prion diseases

None detected.

## Discussion

This is the first multi-site study, in one UK region (NHS Lothian Health Board), assessing the feasibility of enhanced surveillance of CJD in the 65 + population group to detect otherwise unrecognised prion disease. Thirty cases were recruited exhibiting at least one ‘atypical’ neurological feature. These ‘atypical’ features could suggest prion disease, but, are not unique to prion diseases [41–43]. Essentially, a diagnosis of prion disease is based on the clinical pattern, exclusion of other diagnoses, and supportive investigation findings (confirmed by neuropathology). No prion diseases were found.

The *PRNP*-129 genotyping profile of the study cases (32%—MM, 52%—MV, 16%—VV) reflects a profile of the UK general population (37%—MM, 51% -MV, 12%—VV) [11], but different to sCJD and vCJD [16]. This is not surprising, and while incubation and susceptibility to prion disease have been linked to *PRNP*-codon-129 polymorphism, it does not help to distinguish prion from non-prion diseases, and broadly, its role in other neurodegenerative diseases is inconclusive [44, 45]. The duration of illness of the cases was widely varied from 1.1 years to 17.4 years. In itself, the long duration of illness makes a diagnosis of vCJD (median duration, 14 months) or sCJD (median duration, 4 months) improbable [46, 47]. However, unusually long duration in prion disease is recognised [5]. The time interval between symptom onset and referral ranged from 1 to 13 years being shorter on average for the oldest age group (80 +) compared with younger cases. Arguably, this might indicate the possibility that prion disease in the younger 65 + population might have been missed. Another possibility is that a smaller proportion of the younger age range, relative to the older range, might be known to dementia services out-with primary care. However, it is unclear why there was a small number of cases referred after the age of 80 years where the mortality rate from sCJD also declines [16].

The study was, primarily, a feasibility study, trying to determine if it was a successful method of enhanced CJD surveillance in the elderly. While the approach used was well received, there are obvious problems. Firstly, actual numbers recruited did not match predicted numbers due partly to fewer than expected referrals (which does not rule out the possibility of an error in the predicted calculations) and a relatively low participation rate. Secondly, the study relied on the ability of local clinicians to refer eligible cases, who, may not have been able to identify the right cases for various reasons, and if they did, the patients/representative may not have wanted to be referred. Low referral could also have been due to the local clinicians' significant workloads, therefore, no time to speak about the study with potential cases. All these factors are not uncommon in research studies [48–50]. Thirdly, participation was limited both in joining the study (85% referred were eligible) and in full consent (70% agreeing to MRI and 73% with autopsy consent, of which, 8 are alive). Full participation was likely to have been limited by the patient’s/representative’s perception of the study. Precise reasons are unclear, however, perceptions of risks vs. benefits [51] and fear may have had some influence.

The limited recruitment number required the efforts of several study personnel and it is difficult to see how an extension to a larger population would be feasible. The study did not find any missed cases of prion disease, but, given the numbers and methodology involved, it is difficult to draw any firm conclusions on whether there are undetected cases in the elderly. Neuropathology is the final diagnostic determinant but this was available in only 10 cases. An additional 4 cases were missed, which was an unfortunate consequence both due to delays in alerting the study team therefore embalming took place, and the unexpected COVID-19 crisis.

## Conclusion

The results suggest the methodology used is not feasible as a basis for more extended enhanced surveillance for prion disease in the elderly. However, no previously unsuspected cases of prion disease were identified. There were lower than expected referral rates and incomplete recruitment. A review investigating these aspects is being undertaken.

## Data Availability

The data is held by the NCJDRSU as part of their surveillance projects. The NCJDRSU is an internationally recognised World Health Organisation reference centre and European Centre for Disease Control hub for diagnosis of all forms of human prion disease and has substantial expertise in prion disease surveillance and clinical and laboratory research in neurology, neuropathology, brain imaging and biochemical investigations in relation to dementing illness (www.cjd.ed.ac.uk). Enquiries to access the data can be made to NCJDRSU (https://www.cjd.ed.ac.uk/contact-us), which will be considered on a case by case basis in line with the NCJDRSU Data Protection and Security Code of Practice.

## Abbreviations

ACE: Addenbrooke’s Cognitive Examination
AD: Alzheimer’s Disease
AD-NC: Alzheimer’s Disease—Neuropathological change
ARC: Ann Rowling Clinic
BSE: Bovine Spongiform Encephalopathy
CDI: Conformation Dependent Immunoassay
CJD: Creutzfeldt-Jakob Disease
CSF: Cerebral Spinal Fluid
DLB: Dementia with Lewy Bodies
DWI: Diffusion Weighted Imaging
EMAS: Edinburgh Motor Assessment
FAB: Frontal Assessment Battery
FLAIR: Fluid-Attenuated Inversion Recovery
FTD: Frontotemporal Dementia
HADS: Hospital Anxiety and Depression
MRC: Medical Research Council prion disease rating scale
MRI: Magnetic Resonance Imaging
NaPTA: High sensitivity sodium phosphotungstic acid precipitation
NHS: National Health Service
NIA-AA: National Institute on Aging-Alzheimer’s Association
PD: Parkinson’s Disease
PMCA: Protein Misfolding Cyclic Amplification
PRNP: Human Prion Protein Gene
RT-QuIC: Real-time quaking induced conversion
sCJD: Sporadic Creutzfeldt-Jakob Disease
SIB-S: Severe Impairment Battery—Short Form
UK: United Kingdom
vCJD: Variant Creutzfeldt-Jakob Disease
VD: Vascular Dementia
WB: Western blot
